# Persistent chemicals in particulate matter (PM) near a hazardous waste thermal treatment facility

**DOI:** 10.1016/j.apr.2025.102769

**Published:** 2025-10-01

**Authors:** Chuqi Guo, Martine E. Mathieu-Campbell, Thomas Blanchard, Lavrent Khachatryan, Md Abdullah Al-Mamun, Qingzhao Yu, Myron Lard, Oluwafeyikemi Ogunmusi, Brenda Vallee, Wilma Subra, Iriel Edwards, David Malone, Slawo Lomnicki, Stephania A. Cormier, Jennifer Richmond-Bryant

**Affiliations:** a Department of Forestry and Environmental Resources, North Carolina State University, Raleigh, NC, 27695, USA; b Center for Geospatial Analytics, North Carolina State University, Raleigh, NC, 27695, USA; c Department of Biostatistics and Bioinformatics, Rollins School of Public Health, Emory University, Atlanta, GA, 30322, USA; d Department of Oceanography and Coastal Sciences, Louisiana State University and A&M College, Baton Rouge, LA, 70803, USA; e Department of Chemistry, Louisiana State University and A&M College, Baton Rouge, LA, 70803, USA; f School of Public Health, Louisiana State University Health Sciences Center, New Orleans, LA, 70112, USA; g Department of Environmental Sciences, Louisiana State University and A&M College, Baton Rouge, LA, 70803, USA; h Central Louisiana Coalition for a Clean and Healthy Environment, Colfax, LA, 71417, USA; i Subra Company, New Iberia, LA, 70563, USA; j Shreveport Green, Shreveport, LA, 71104, USA; k Louisiana Department of Wildlife and Fisheries Region 3, Pineville, LA, 71360, USA; l Department of Biological Sciences, Louisiana State University and A&M College and Pennington Biomedical Research Institute, Baton Rouge, LA, 70803, USA

**Keywords:** Open burn/open detonation, PM_2.5_, Environmentally persistent free radicals, Thermal treatment, Military waste, Community-engaged research

## Abstract

Colfax, an overburdened community in central Louisiana, hosts the last commercially-operated open-burn/open-detonation (OB/OD) hazardous waste thermal treatment facility in the United States. Until December 2023 when their permit disallowed OB/OD, the facility processed military waste, fireworks, propellants, soils excavated from Superfund sites, and other hazardous materials. This community-engaged study measured ambient fine particulate matter (PM_2.5_), environmentally persistent free radicals (EPFRs), polychlorinated dibenzo-p-dioxins and dibenzofurans (PCDD/Fs), and metals using two high-volume PM_2.5_ samplers deployed 1.2 mi and 9.0 mi from the facility from April, 2022 through February, 2023. Elevated PM_2.5_ concentrations were recorded at both sites during spring and summer 2022. EPFR concentrations increased during fall and winter, coinciding with increases in PCDD/F but in contrast to PM_2.5_. Similarities between the 1.2 mi and 9.0 mi sites for both PM_2.5_ and EPFRs suggest a common emission source influencing concentrations at both sites. Regularized linear regression analyses indicated that EPFRs were significant predictors of PM_2.5_ at both sites, with a markedly stronger effect at the 9.0 mi site than at 1.2 mi. Among the metals, Zn was consistently the strongest and most significant predictor of EPFRs and PM_2.5_ across both sites and seasons, supported by both moderately high Pearson correlations and Elastic Net coefficients. Through this study, we aim to provide crucial information on exposure risks from an OB/OD facility with the goal of empowering exposed community members for mitigating their risks.

## 1. Introduction

**Fig. 1.**
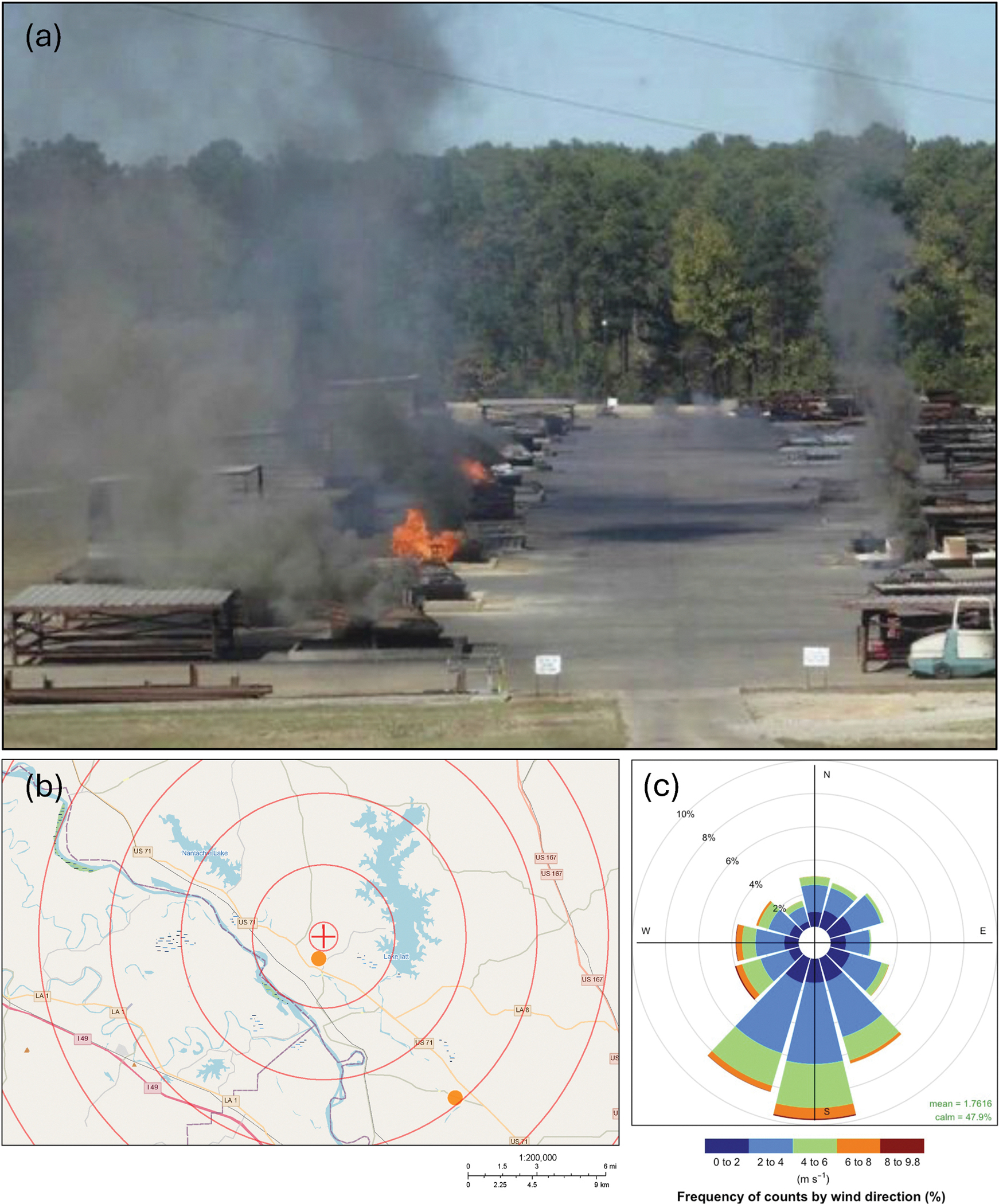
(a) Photograph of the open burn/open detonation hazardous waste thermal treatment site in operation (photograph courtesy of the Louisiana Department of Health). (b) Map illustrating location of HiVol samplers (orange dots) and the TT facility (red cross-hair). (c) Wind rose illustrating the wind speed and direction during the period March 2022–March 2023. Mean wind speed: 1.76 m/s; calm winds 47.9 % of the time.

Colfax, located in central Louisiana, USA, is a rural small town with a population of 1442 as of 2023 (U.S. Census Bureau, 2023). The demographic distribution consists of 61.1 % Black or African American residents and 33.1 % White residents. The median individual income is $20,192, and approximately 35 % of the population lives below the poverty line (U.S. Census Bureau, 2023). These demographic characteristics indicate the community’s structural vulnerability. Colfax is home to a Resource Conservation and Recovery Act thermal treatment (TT) facility (Fig. 1, Fig. S1 in the Supporting Information), which was permitted to process waste through an open burn/open detonation (OB/OD) operation since 1985 (LDEQ, 2023a, 1985).

Many Colfax residents were not aware of the TT facility’s existence until 2014 (Richmond-Bryant et al., 2022), when the facility began accepting explosive military waste from Camp Minden, a Louisiana National Guard site which was the site of a massive explosion in 2012. After the facility began to accept waste from Camp Minden for OB/OD, operations changed from sporadic to daily (M-F, 8–9 h per day). The waste streams treated in this facility include military ordnances; cylinders; explosives; propellants; and other hazardous wastes including those from Superfund sites, unspent fireworks, and ammonium perchlorate. These materials also contain metals and organohalogens (Alternatives for the Demilitarization of Conventional Munitions, 2019; Aurell et al., 2017; LDEQ, 2020). As a result, residents observed thick black smoke, noise, and damage to their property. The TT facility is the only commercially operated facility of its kind in the country. The most recent permit of the facility caps explosive waste destruction at 561,700 pounds per year and bans open burning, although that requirement is on hold (as of January 2025) pending a lawsuit (LDEQ, 2023a).

Operations at the TT facility have been followed by health conditions that may have been related to exposure to toxic gases and particulate matter (PM). Many residents within The Rock community (a historically Black enclave located at the southern border of the facility) and Colfax have described experiencing symptoms and diagnoses related to thyroid disease, respiratory disease, cardiovascular disease, skin damage, and cancer following the expansion of operations in the mid-2010s (Richmond-Bryant et al., 2022). Exposure to the PM and PM associated pollutants, including Environmentally Persistent Free Radicals (EPFRs), polychlorinated dibenzo-p-dioxins and dibenzofurans (PCDD/Fs), and metals, has been associated with many of the health outcomes community members have reported (Aryal et al., 2023; Bertazzi et al., 2001; Harmon et al., 2021; Hogervorst et al., 2006; Mahne et al., 2012; Pelucchi et al., 2009; Saravia et al., 2013; US EPA, 2022a; Wang et al., 2022; Zhang et al., 2020), thus these pollutants are the target analytes of this study.

EPFRs are an emerging class of contaminants that can form through multiple pathways, including naturally occurring sources such as biomass and coal (Bährle et al., 2015; Liu et al., 2014), and as products of thermal processes like fly ash and soot (Wang et al., 2018; Zhao et al., 2019). Among these, formation during combustion is especially important, as organic pollutants interact with metals in the cooler zone (<600 °C) of a combustion system to generate persistent radicals (Cormier et al., 2006; Lomnicki et al., 2008a; Vejerano et al., 2018), and this pathway is expected to be the primary source near the facility. Essentially, EPFRs are PM with a free radical at the surface. EPFRs induce oxidative stress by producing reactive oxygen species (ROS) in biological systems (Ayres et al., 2008; Dellinger et al., 2001a; Khachatryan et al., 2011). Recent research has demonstrated that exposure to EPFRs induces cardiopulmonary injury (Aryal et al., 2023; Mahne et al., 2012), resulting in pulmonary dysfunction (Harmon et al., 2021), airway hyperresponsiveness (Balakrishna et al., 2011), and cardiovascular dysfunction (Lord et al., 2011). Preliminary data from a cohort study have shown that exposure to indoor dust containing oxygen-centered EPFRs is correlated with wheeze in children (Sly et al., 2019).

PCDD/Fs are toxic combustion byproducts generated from the burning of hazardous waste. Detonations of ammonium perchlorate and munitions were declared in many of the burn logs (LDEQ, 2023b, 2023c, 2023d, 2022a, 2022b, 2022c, 2022d) released by the facility, and perchlorates are known to be present in some munitions. When chlorinated organics are present during combustion, PCDD/Fs can be produced under the same conditions in which EPFRs are formed (Chen et al., 2020). These conditions occur during TT operations (Cruz et al., 2012; Liu et al., 2020; Walsh et al., 2010). It should be noted that the burn logs were only released after residents filed complaints with LDEQ (Richmond-Bryant et al., 2022, 2024). Therefore, reported emissions data likely underestimate the actual waste destroyed and resulting emissions. Short-term exposure to high concentrations of PCDD/Fs could lead to skin lesions (DeVito et al., 2024; Saurat et al., 2012; Sorg and Saurat, 2023) and altered liver function (Czepiel et al., 2009), and chronic exposures have been linked to damage to the immune (Halperin et al., 1998; Vos et al., 1973), endocrine, and reproductive systems (Kogevinas, 2001; Mocarelli et al., 2008); developmental disruption (Carney et al., 2006; Yoshioka and Tohyama, 2019); thyroid dysfunction (Baccarelli et al., 2008; Warner et al., 2020); and cancers (Manz et al., 1991; Xu et al., 2016). Previous studies have demonstrated that EPFRs are precursors or intermediates to form PCDD/Fs under the same thermal conditions (Liu et al., 2021; Lomnicki and Dellinger, 2003; Vejerano et al., 2021).

Metals can be present in munitions waste streams (Alternatives for the Demilitarization of Conventional Munitions, 2019; Aurell et al., 2017). Lead (Pb), aluminum (Al), antimony (Sb), copper (Cu), nickel (Ni), zinc (Zn), chromium (Cr), tungsten (W), beryllium (Be), and arsenic (As) are present in ammunition (Alternatives for the Demilitarization of Conventional Munitions, 2019; Datta et al., 2017; Ryu et al., 2007; Sanderson et al., 2018), and vanadium (V), barium (Ba), manganese (Mn), iron (Fe), Cr, Cu, Zn, As, and Pb are commonly found in fireworks (Do et al., 2012). Excessive exposure to these metals could result in several acute and chronic toxic effects on various organ systems, including the skin, gastrointestinal, renal, immune, and nervous systems (Balali-Mood et al., 2021; Deng et al., 2019; Lech and Sadlik, 2017; Mishra, 2009). Be and Cd are classified by the International Agency for Research on Cancer (IARC) as known carcinogens (IARC, 1993) and inorganic Pb is classified as a probable carcinogen based on evidence for effects in humans by IARC (IARC, 2006) and the U.S. Environmental Protection Agency (EPA) Integrated Risk Information System (US EPA, 2004).

In response to requests from the community coalition formed in Colfax, the Central Louisiana Coalition for a Clean and Healthy Environment, we created the first air monitoring program in and around Colfax. This community-engaged study was developed based on community interviews (Richmond-Bryant et al., 2024) and community input. The objective of this study is to determine the presence and extent of EPFRs, PCDD/Fs, and metals in ambient air PM samples in the vicinity of the Colfax TT facility and to explore the associations among these substances, which could indicate that the TT facility is a common source. Concentration trends were compared, and associations among measured pollutants were investigated. Collected data may provide confirmation to residents that their symptoms are consistent with potential health risks of EPFRs, PCDD/Fs, and metals identified in PM samples, and thus enhance residents’ understanding of their exposures. As a result, this study is expected to promote community empowerment and engagement and to better understand the extent of distributive environmental injustices.

## 2. Materials and methods

### 2.1. Sample collection

PM_2.5_ samples were collected at two sites identified at 1.2 mi SSW and 9.0 mi SE of the TT facility’s burn site between April 1, 2022 and March 16, 2023. Sample site selection was informed by an oral history study conducted in the Colfax community (Richmond-Bryant et al., 2022). Samples were generally collected on a weekly basis. Concentrations were calculated for one-week periods using time-weighted averaging. We deployed two high-volume (HiVol) PM_2.5_ samplers (HiVol 3000, American EcoTech) and employed quartz fiber (QF) filters (TissuQuartz^™^, 20.32 cm × 25.40 cm, Pall) as the collection substrate. The samplers were operated at a constant volumetric flow rate of 67.8 m^3^/h, and the instruments were calibrated after initial assembly and then every 3 months at 60, 70, and 80 m^3^/h flow rates. The greased collection shims, used to capture larger particles, as well as the filter cassettes and supporting frames holding the QF filters, were cleaned weekly between sample collections. Additionally, other parts of the instruments were cleaned and maintained every three months, prior to flow rate calibration, as recommended by the manufacturer’s manual.

“Burn logs” are operational records kept by the TT facility to track their inventory and burn activities. Burn logs contain information about material type, quantity, and timeframe when waste was combusted. The facility is required to release the burn logs to LDEQ from a specific date if a resident complaint was filed on that day (LDEQ, 2020). The waste burning/detonation data from April, 2022 to February, 2023 were reviewed, and the quantities of gross and explosive material destroyed were extracted from the burn logs for the estimation of PM_2.5_ emissions. A total of 74 days of records were available during the 2022–2023 sampling campaign, and hourly burn logs provided classification codes and compatibility groups for each type of waste as defined in the code of federal regulations (CFR) Title 49 Subtitle B Chapter I Subchapter C Part 173 Subpart C (The Federal Register OFR, 1990). The compilation of air pollutant emissions factors (AP-42), that has been produced by the U.S. EPA since 1972, provides emissions factors (EF) for various sources of air pollutants, including PM_2.5_, for both stationary points and area sources (US EPA, OAR, 2016). The EFs are expressed as the mass of the pollutant emitted per mass of fuel. We used AP-42 Chapter 15 on Ordnance Detonation, AP-42 Chapter 2 on Solid Waste Disposal, and more recent studies on emissions from detonation of munitions for this calculation (Aurell et al., 2015; Gullett et al., 2016). PM_2.5_ emission from explosive and non-explosive wastes treated at the TT facility were estimated by the quantity of each waste type and their corresponding EFs (Aurell et al., 2017).

### 2.2. Meteorological data

Analysis of wind data was performed using R (v. 4.4.2) with the packages *openair*, *worldmet*, and *tidyverse*. Data were imported using the *importNOAA* function in *worldmet*, which retrieves data from the National Oceanographic and Atmospheric Administration (NOAA) Integrated Surface Database (ISD). Data were retrieved for 2022 and 2023 for station 722487–13935, which corresponds to the Alexandria, LA airport and is the closest NOAA-operated station to the Colfax, LA study site (31.5 km to the southeast). Data were then selected for the sampling period of April 1, 2022–February 23, 2023, and wind roses were created using the *windRose* function. Output includes data for predominant winds and for calm winds, with a wind speed of 0 m/s and a direction of 0°.

### 2.3. Chemical analysis

Prior to initiating sample collection, all QF filters were prebaked at 450 °C for 6 h to remove possible VOCs, then equilibrated for 24 h before pre-weighing. To ensure integrity of the analyte, all pre-cleaned QFs were stored under − 18 °C. Samples were stored in an electric cooler during transportation from the field site to the laboratory. Post-collection, QF filters were again stored under − 18 °C to avoid sample loss. Two QF field blank filters were created before the sample collection campaign by loading the filters into on-site instruments, leaving them in place for 1 min without the sampler pump running, and then retrieving them. Post-collection QF filters were weighed to determine the mass of the collected PM_2.5_. The QF filters were then sectioned into four segments of equal area using ceramic scissors for analysis for EPFRs, PCDD/Fs, and metals. The fourth section was reserved for potential future investigations. To consistently report the weekly PM_2.5_, EPFR, and metal concentration levels, weighted weekly averages were calculated for all the raw data from the 1.2 mi and 9.0 mi sites during the period from April 1, 2022 to February 23, 2023. Data from February 24, 2023 to March 15, 2023 were excluded from the analysis due to the significant mass accumulation of pollen during the last sampling weeks.

Electron paramagnetic resonance (EPR) spectroscopy (EMX-20/2.7 EPR spectrometer (X-band), Bruker) was utilized for measuring radical signals in samples. From a single quadrant of a post-collection QF filter, three strips measuring 0.5 cm by 8.9 cm were excised from the top, center, and bottom, and then they were further divided into small fragments and packed into 0.5 cm outer diameter (OD) EPR tubes for spectral measurements. They were measured separately, averaged, and the standard deviation of EPFR concentration (number of radicals/g of sample) was calculated. To improve the spectral background measurements and more finely resolve the metal spectra, a liquid nitrogen (N_2_) Dewar flask was positioned outside the EPR tubes during measurements. Sample signals were calibrated against a 30 μg 2,2-diphenyl-1-picrylhydrazyl (DPPH) standard reference (Thermo Scientific). The parameters for EPR measurements were as follows: microwave frequency of 9.629 GHz; power of 2.03 mW; 4.0 G modulation amplitude; 100 G sweep width; 167.77 s sweep time; 40.96 ms time constant (corresponding to a 163.84 ms conversion time); 3.56 × 10^4^ receiver gain; and three scans were performed. Finally, ambient EPFRs concentrations were recalculated in units of radicals/m^3^ of air by dividing the EPFR concentration in units of radicals/g by the PM_2.5_ concentrations in units of g/m^3^.

Because the detonation of ammonium perchlorate was declared in some of the burn logs (LDEQ, 2023b, 2023c, 2023d, 2022a, 2022b, 2022c, 2022d) and perchlorates are found in some munitions (Alternatives for the Demilitarization of Conventional Munitions, 2019), PM_2.5_ samples from the following collections, along with a field blank, were analyzed for PCDD/Fs by Eurofins Environmental Testing (Knoxville, TN): April 8–15, 2022, June 17–24, 2022, July 1–15, 2022, September 17–24, 2022, November 19–26, 2022, and December 16–26, 2022. Samples were selected for PCDD/Fs analysis to include a range of ammonium perchlorate treatment quantities (Table S1, Supporting Information). U.S. EPA Standard Method TO-9A was followed for the PCDD/F high resolution gas chromatography-high resolution mass spectrometry analysis of 17 congeners: 2,3,7,8-TCDD; 1,2,3,7,8-PeCDD; 1,2,3,4,7,8-HxCDD; 1,2,3,6,7,8-HxCDD; 1,2,3,7,8,9-HxCDD; 1,2,3,4,6, 7,8-HpCDD; OCDD; 2,3,7,8-TCDF; 1,2,3,7,8-PeCDF; 2,3,4,7,8-PeCDF; 1, 2,3,4,7,8-HxCDF; 1,2,3,6,7,8-HxCDF; 2,3,4,6,7,8-HxCDF; 1,2,3,7,8, 9-HxCDF; 1,2,3,4,6,7,8-HpCDF; 1,2,3,4,7,8,9-HpCDF; and OCDF. A laboratory control sample, a method blank sample, and a field blank sample were employed in the analysis, and the results were compared to the method detection limit and the recovery acceptance limit.

The total toxicity of PCDD/Fs is usually evaluated by toxic equivalency (TEQ), which is the sum of the concentration of the i-th PCDD/F congener (C_i_) weighted by the congener’s toxic equivalency factor (TEF_i_) (Safe, 1990; U.S.EPA, 2010):

$$TEQ=\sum_{i=1}^{n}C_{i}\times TEF_{i}$$

Each TEF is a consensus-determined weighting factor between 0 and 1 that accounts for each congener’s toxicity relative to the most toxic form of PCDD/Fs, 2,3,7,8-tetrachlorodibenzo-p-dioxin (2,3,7,8-TCDD), which is assigned a TEF of 1 (U.S.EPA, 2010). U.S. EPA recommended the use of the consensus TEF values published in 2005 by the World Health Organization (WHO) (U.S.EPA, 2010; Van den Berg et al., 2006). In October 2022, new consensus TEFs, based on a larger body of data analyzed via Bayesian dose-response modeling and meta-analysis, were assigned to PCDD/Fs by a WHO-convened expert panel (DeVito et al., 2024). In this study, TEQ was calculated for each sample date based on the 2022 TEFs (DeVito et al., 2024).

The PM_2.5_ QF filter samples were acid digested for metal analysis by Inductively Coupled Plasma Atomic Emission Spectroscopy (ICP-AES). The acid digestion method was adapted from U.S. EPA Standard Method 3050B (US EPA, 2019). Briefly, a quadrant (11.4 cm × 8.9 cm) of a PM_2.5_ QF filter was weighed and pre-digested overnight in 12.5 mL of concentrated nitric acid (HNO_3_) (Aristar Plus^®^, VWR 87003–261) at room temperature. The QF filter blanks and samples were then acid digested using a BD50 (Seal Analytical) programmable digestion system at 50 °C for 2 h and then 110 °C for 16 h. Cleaned glass funnels were used to reflux the solution and prevent evaporation. Post-cooling, 4 mL of 30 % H_2_O_2_ (Aristar^®^ Ultra, VWR) was added, followed by another 2-h hold at 110 °C, and then 5 mL of HCl was added with a 30-min hold at 110 °C. After digestion, samples were evaporated down to approximately 5 mL, diluted with deionized water (≥18.2 MΩ•_⋅_cm) to 60–65 mL solution, weighed, mixed, and filtered for ICP-AES analysis. Details of the analysis and quality assurance/quality control are given in Text S1 (Supporting Information). The 18 metals analyzed in this study were silver (Ag), As, Al, Ba, Be, cadmium (Cd), cobalt (Co), Cr, Cu, Fe, magnesium (Mg), Mn, Ni, Pb, Sb, V, tungsten (W), and Zn. All metal samples were analyzed with recovery standards (indium and neodymium) and an internal standard (yttrium).

Particle surface area was estimated from PurpleAir concentrations retrieved from collocated sensors at the 1.2 mi and 9.0 mi sampling sites. PurpleAir reports particle counts detected every 2 min for 0.3 μm, 0.5 μm, 1.0 μm, 2.5 μm, 5.0 μm, and 10.0 μm particles. Surface area, $S_{i}$, of an individual particle i was calculated as $S_{i}=\pi d_{i}^{2}$ , where $d_{i}$ = diameter of particle i. Surface area for each particle measurement was bias corrected using a multiple linear regression correction factor to account for humidity and temperature in the Southeastern U.S. (Mathieu-Campbell et al., 2024). Bulk surface area, S, was calculated as the sum across particles, $S=\pi \sum_{i}n_{0.3}0.3^{2}+n_{0.5}0.5^{2}+n_{1.0}1.0^{2}+n_{2.5}2.5^{2}$, where n represents particle count at each particle diameter.

### 2.4. Statistical analysis

Summary statistics were calculated at the beginning of the analysis to identify general patterns in the data. Weekly data were analyzed for PM_2.5_, EPFR, and metal concentrations. Summary statistics were calculated for PCDD/Fs during the weeks when samples were analyzed. Data manipulation was performed in R (v. 4.2.3) using the packages *dplyr*, *lubridate*, *tidyr*, *RColorBrewer*, *ggplot2*, and *tidyverse*. Calculations were performed primarily with the R library *stat*. Autoregressive integrated moving average (ARIMA) modeling was performed to deduce time series behavior (Text S2, Supporting Information).

Correlations and regressions were both calculated, because correlation explains variability in a relationship between two variables while regression calculates how a change in one variable produces a corresponding change in another variable (i.e., slope). Statistically significant regression slopes sometimes correspond to high correlations, but slopes can be significant even if the relationship has substantial scatter. Pearson correlations (ρ) between concentrations of PM_2.5_, EPFRs, and metals retrieved from the HiVol samplers’ filters and particle surface area retrieved from the PurpleAir data files and averaged over matching 1-week time periods were tested using the *stat* and *corrplot* libraries in R. Pearson correlations were also calculated for monthly emissions estimates with 1-month time-averaged concentrations of PM_2.5_ and EPFRs. Regularized linear regression methods were then applied to investigate the strength of relationships of PM_2.5_ and EPFRs with all measured metal concentrations using the weekly data at each site with the R library *glmnet*. Regularized linear regression methods are considered exploratory, but they permit identification of potentially important relationships. Whereas ordinary least squares regression assumes that independent variables are not correlated with one another, regularized linear regression methods have the capacity to include multiple correlated independent variables by constraining the residual sum squares (RSS) to some condition (Hastie et al., 2009). The Least Absolute Shrinkage and Selection Operator (LASSO) constrains the RSS to the sum of the absolute value of the model coefficients, weighted by a penalty factor, λ. A Ridge Regression constrains the RSS to the sum of the squares of the model coefficients, weighted by λ, but does not reduce dimension of the model. The parameter α is a multiplier varying between 1 and 0 that defines these constraints. The Elastic Net uses a combination of the two approaches, minimizing the number of variables while optimizing the prediction coefficients with an α between 0 and 1. For this reason, Elastic Net output was used in this study, and trial and error was used to find the α that minimized RSS. The *cv.glmnet* function in R was used to identify the value of λ that minimized the mean cross-validated error in the Elastic Net using the optimized α. The following versions of the models were run for the 1.2 mi and 9.0 mi sites:

(1) $$EPFR_{0}=\beta_{0}+\beta_{-1}EPFR_{-1}+\sum_{i}\beta_{i}Metal_{i,0}$$

(2) $$PM_{2.5,0}=\beta_{0}+\beta_{-1}PM_{2.5,-1}+\beta_{e,0}EPFR_{0}+\sum_{i}\beta_{i}Metal_{i,0}$$

We also tested lag-1 metals (shown with the “−1” subscript) terms in the models to evaluate whether previous-week EPFR, PM_2.5_, and metal concentrations influenced current-week levels. We found little difference between the models with the lag-1 metals and those with only the lag-0 metals. For this reason, we present the models without the lag-1 metals for parsimony. Models were not developed for PCDD/Fs given that the small number of samples in those datasets compared with the number of variables creates an ill-posed model. Similarly, regularization models were developed for a full year because models with a number of samples that is similar in size to the number of variables are unstable.

## 3. Results and discussion

**Fig. 2.**
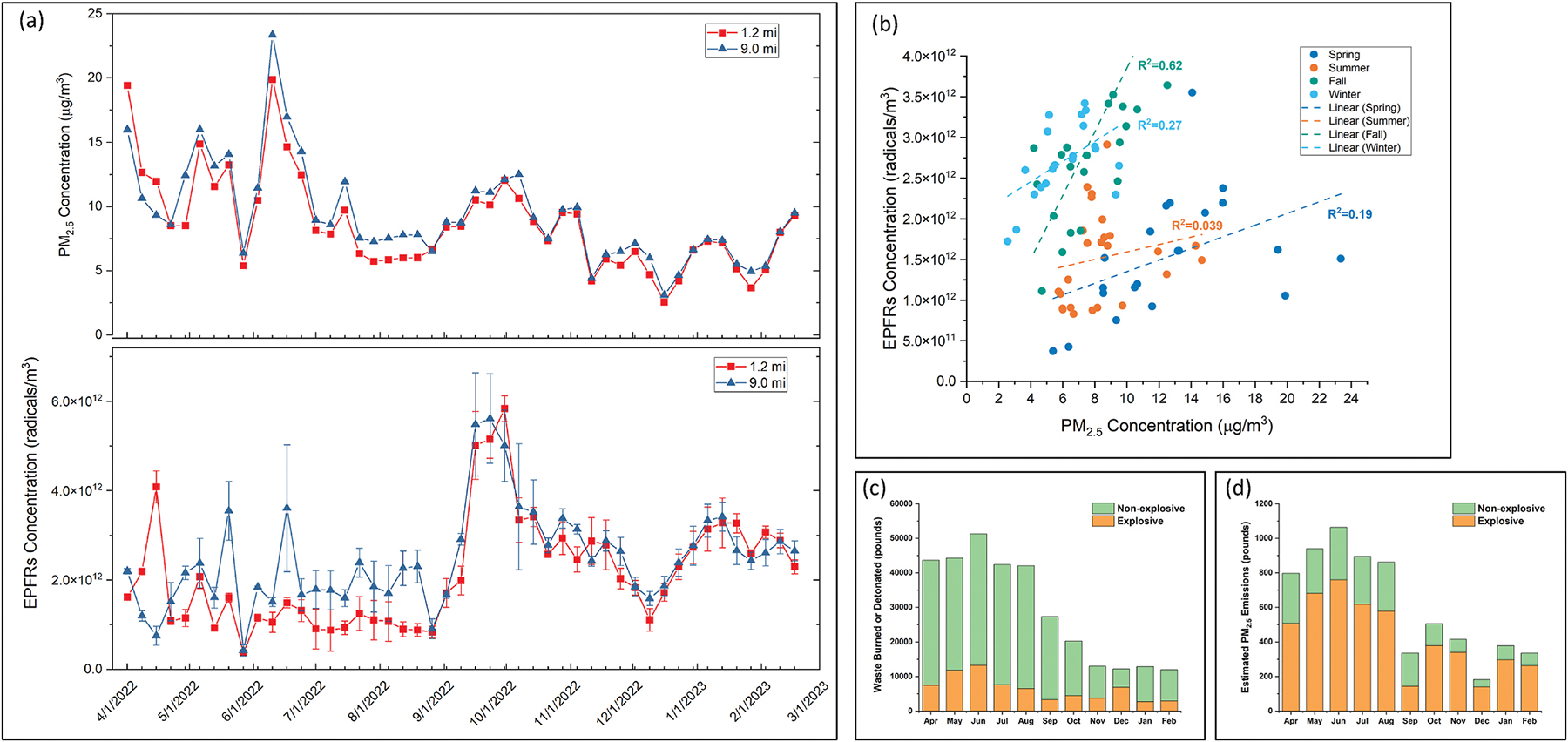
(a) PM_2.5_ and EPFR concentrations measured with HiVol samplers from April 1, 2022, to February 23, 2023. (b) Scatter plots of EPFR vs. PM_2.5_ concentrations by season: spring (April 1–June 16, 2022), summer (June 17–September 15, 2022), fall (September 16–December 15, 2022), and winter (December 16, 2022–February 23, 2023). (c) Monthly amounts of explosive and non-explosive waste burned or detonated, and (d) estimated monthly PM_2.5_ emissions corresponding to the waste treated during the same period. Data in panels (c) and (d) are based solely on burn logs provided by the facility to LDEQ.

The time series of the weighted weekly PM_2.5_ concentrations from the 1.2 mi and 9.0 mi sites during the period from April 1, 2022 to February 23, 2023 displayed higher concentrations in the spring and summer compared with the fall and winter (Fig. 2 (a)). Weekly-averaged PM_2.5_ concentration ranged from 2.57 μg/m^3^ to 19.88 μg/m^3^ at the 1.2 mi site and from 3.10 μg/m^3^ to 23.35 μg/m^3^ at the 9.0 mi site, with averages of 8.67 μg/m^3^ and 9.37 μg/m^3^. The highest PM_2.5_ levels for both sites were detected during the week of June 10 to June 16, 2022. PM_2.5_ concentrations were similar at both sites, which shared time-series characteristics of a 1-lag autoregression. Our results revealed elevated PM_2.5_ concentrations at both the 1.2 mi and 9.0 mi sites during the spring and summer, with similar patterns observed at both locations. Wind direction was investigated as a key meteorological factor influencing pollutant distribution. The wind rose for the entire sampling campaign indicates a high percentage of calm winds (47.9 % of the time), during which both sites could potentially be influenced by facility emissions. At other times, however, the 9.0 mi site functioned as an upwind site. Annual, seasonal and monthly wind roses are shown in Figs. S2–S4 (Supporting Information). The similar PM_2.5_ patterns observed at both sites suggest a common emission source. In an open field under calm wind conditions, it would be anticipated that the 1.2 mi site would display higher concentrations. However, complex dynamics related to plume rise and development and action of trees near the 1.2 mi site acting as a wind break may contribute to the observation of similar concentrations at both sites (Briggs, 1982; Gifford, 1982; Lin et al., 2006). The elevated PM_2.5_ concentrations at the 9.0 mi site could be attributed to several factors, such as the plume from the burn site being too hot to settle at the 1.2 mi site, partial blockage of the plume by trees preventing the particle capture by the sampler, and other agricultural or traffic-related activities affecting the PM_2.5_ concentration measured at 9.0 mi site.

The comparison of the HiVol sampler PM_2.5_ concentrations with the PM_2.5_ emissions (Fig. 2) estimated from the burn logs revealed a similar annual trend. The quantity of materials burned or detonated and the estimated PM_2.5_ emissions appeared to be highest in June, 2022, coinciding with the peak PM_2.5_ concentrations recorded by the HiVol samplers. Likewise, reduced overall operations in the fall and winter corresponded with lower PM_2.5_ emissions. Although explosive waste accounted for a smaller fraction of the total waste, it contributed more than half of the PM_2.5_ emissions based on emissions factors estimated for open burning of munitions (Aurell et al., 2015). According to annual reporting, the information in burn logs represents 38.8 % of explosive waste and 42.0 % of nonexplosive waste burned at the TT facility (LDEQ, 2024, 2023e). Annual air reports from the facility further indicate that the total volume of explosive wastes treated in 2017 was approximately 1.5 and 2 times higher than in 2022 and 2023, the years during which our sampling was conducted (LDEQ, 2024, 2023e, 2018). This discrepancy in treatment volume should be considered when interpreting results, as the current measurements may underestimate the historical peak levels of pollutant emissions and associated community exposures.

EPFR concentrations (Fig. 2) at the 1.2 mi site ranged from 3.72 × 10^11^ radicals/m^3^ to 5.84 × 10^12^ radicals/m^3^, with an average of 2.15 × 10^12^ radicals/m^3^. These values correspond to 5.31 × 10^16^ to 7.10 × 10^17^ radicals/g, with an average 2.89 × 10^17^ radicals/g. At the 9.0 mi site, EPFR concentrations ranged from 4.26 × 10^11^ radicals/m^3^ to 5.61 × 10^12^ radicals/m^3^, with an average of 2.48 × 10^12^ radicals/m^3^. These values correspond to 6.47 × 10^16^ to 6.01 × 10^17^ radicals/g, with an average 2.98 × 10^17^ radicals/g. The highest EPFR concentrations were detected during fall 2022. Both sites shared the same time-series characteristics, with a 1-lag autoregression. The similar patterns observed at both sites suggest influence from a common source, which may include both emissions from the facility and contributions from the regional background, during the majority of the 2022–2023 sampling period. The EPFR concentration range observed in this study is generally higher than the ambient PM_2.5_-associated EPFR levels reported in several U.S. studies, which were conducted in urban settings where traffic emissions and residential activities were the primary sources (Dellinger et al., 2001b; Runberg et al., 2020; Squadrito et al., 2001). The g-factor of EPFRs, which indicates the intrinsic properties of an electron, provides information about the radical type, such as carbon-centered (where an unpaired electron is located on a carbon atom), oxygen-centered (where an unpaired electron is located on an oxygen atom), or a radical containing a heteroatom, for example, an oxygen atom adjacent to a carbon-centered aromatic radical (Dellinger et al., 2007), with each having a specific g-factor. Although various factors influence the activity of carbon- and oxygen-centered radicals under ambient conditions, both types are considered hazardous due to their ability to generate ROS (Harmon et al., 2018; Zhao et al., 2024). The g-factors of the EPFRs measured at both sites throughout the one-year sampling campaign ranged from 2.0028 to 2.0036 at the 1.2 mi site and 2.0029 to 2.0036 at the 9.0 mi site. These findings suggest the presence of a complex mixture of carbon-centered radicals, oxygen-centered radicals, and carbon-centered radicals with an adjacent oxygen, the latter of which has been reported to exhibit a g-factor range from 2.003 to 2.004 (Dellinger et al., 2007, 2011; Lomnicki et al., 2008b; Odinga et al., 2020; Xu et al., 2019). Similar types of EPFRs with comparable g-factors have been detected from soot (Dellinger et al., 2000; Jia et al., 2020); smoke from cigarettes and e-cigarettes (Hasan et al., 2020; Valavanidis and Haralambous, 2001); ambient PM_2.5_ potentially associated with coal combustion, traffic, and secondary sources (Qian et al., 2020; Yadav et al., 2016); Superfund site soils (Cruz et al., 2012); crude oil (Guedes et al., 2006); and petroleum asphaltenes (Montanari et al., 1998).

Fig. 2 (b) shows the seasonal relationships between PM_2.5_ and EPFR concentrations. The strength of the correlations varied notably by season. A relatively strong positive correlation was observed in fall (R^2^ = 0.62), which coincided with the time when EPFRs concentrations were highest. Conversely, correlations were weaker in the spring (R^2^ = 0.19) and summer (R^2^ = 0.039) when PM_2.5_ concentrations were higher. Variation in correlation between PM_2.5_ and EPFRs might have been related to variation in the materials burned or detonated at the TT facility over time and variation in meteorological conditions over the course of the year.

**Fig. 3.**
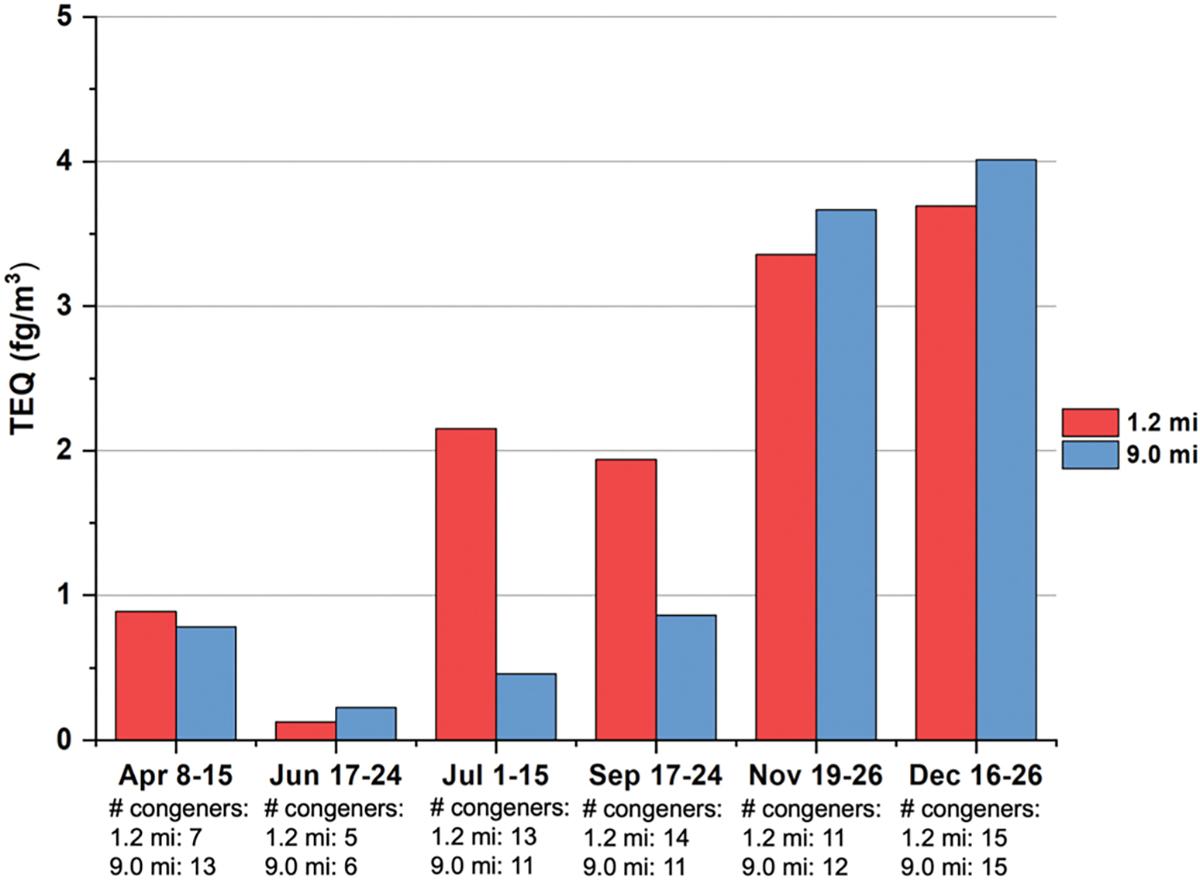
TEQ (calculated as the product of TEF and congener concentration) at two sites during selected weeks. All samples were collected in 2022. Individual congeners’ concentrations, scaled by their TEF, are shown in Fig. S5 (Supporting Information).

Since combustion of ammonium perchlorates was declared in some of the burn logs, several samples were selected for PCDD/F analysis. Fig. 3 presents the TEQ for each collection event, and the contributions of individual congeners to the TEQ are shown in Supporting Information Fig. S5. A summary for the quantity of ammonium perchlorates declared in the burn logs coinciding with the sample collection periods is provided in Table S1 (Supporting Information). TEQ of these measured collections from two sites ranged from 0.1288 to 4.0095 fg/m^3^. The detection of PCDD/Fs in April and September, when there was no declaration of ammonium perchlorate in the burn logs, indicated other sources in the treated waste, potentially munitions. Higher TEQ values were observed later in the entire sampling period, when EPFR concentrations were also higher. Considering the intermittent release of the PCDD/Fs and the strong dependence of their detection on meteorological conditions, particularly wind direction, the TEQ over a period may not accurately represent short-term exposure levels. Recurrent short-term exposure to elevated PCDD/F concentrations over the past 40 years, particularly during periods when the facility processed larger volumes of waste than in the sampling year, continues to pose a significant health risk to residents of The Rock and Colfax communities. Supporting this concern, a large-scale study involving 2 million individuals reported that inhalation exposure to PCDD/Fs may be associated with reduced life expectancy (Dziubanek et al., 2017).

Similarity and differences were both observed for the number and types of congeners, including the most toxic ones, among collections (Fig. S5, Supporting Information). This could be due to the types of waste materials detonated at various time periods. In addition to waste explicitly declared to contain ammonium perchlorate, destroyed munitions may also contain unknown quantities of ammonium perchlorate or potassium perchlorate as accelerants (Aurell et al., 2017; Brandhuber et al., 2009). No other industrial activities have been identified in the area. 1,2,3,4,6,7,8-HpCDD and 1,2,3,4,7,8-HxCDF, which have TEFs of 0.05 and 0.3, contributed the most to the TEQ on most collections (DeVito et al., 2024). The trend in the most frequently detected congeners was consistent between the two sites, indicating common sources of PCDD/F emissions and supporting our assertion that pollutants from the facility can travel at least 9 miles. Cormier et al. (2006) (Cormier et al., 2006) and Vejerano et al. (2018) (Vejerano et al., 2018) presented mechanisms for formation of EPFRs, which then may serve as precursors or intermediates (Sidhu and Dellinger, 1997; Sidhu et al., 1995) in the formation of PCDD/Fs when organic chlorinated compounds such as perchlorates are combusted and then cool and undergo surface-mediated reactions (Khachatryan et al., 2007).

**Fig. 4.**
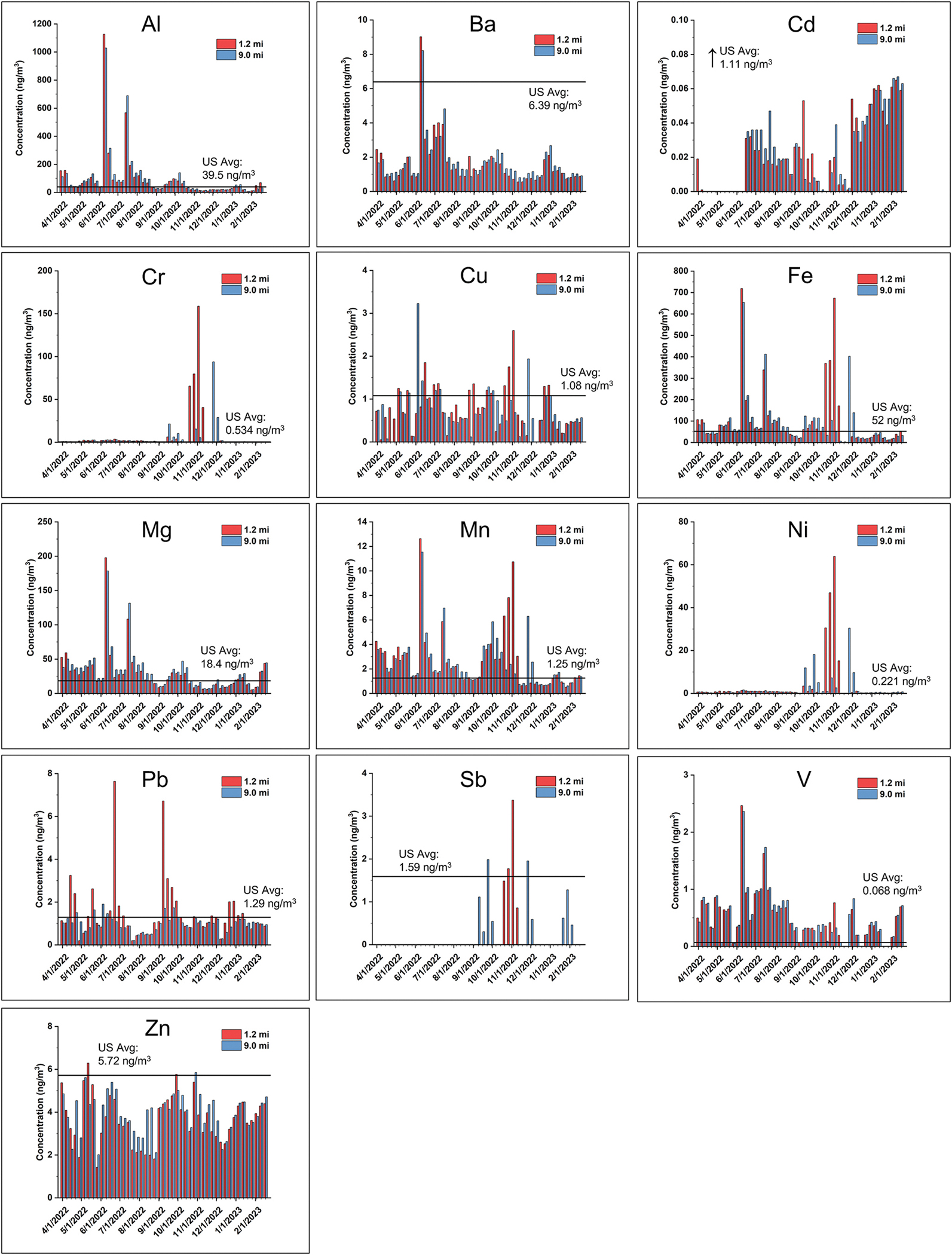
Weekly averaged metal concentrations in PM_2.5_ collected using the HiVol samplers from April 1, 2022 to February 23, 2023. “US Avg” indicates the 50th percentile of average PM_2.5_ metal concentrations in all U.S. counties with speciation monitors (2022) and is shown by a horizontal line on each panel.

Among the 18 metals analyzed in the weekly averaged HiVol PM_2.5_ samples, 13 were detectable in this study (Al, Ba, Cd, Cr, Cu, Fe, Mg, Mn, Ni, Pb, Sb, V, and Zn) from both sampling sites (Fig. 4). Ag, As, Be, Co, and W were analyzed but were below the method detection limit (Table S2, Supporting Information) for all samples. Al, Fe, and Mg, which are found in high concentrations in soil (Smith et al., 2013), were the most abundant metals in HiVol PM_2.5_ samples. All of the analyzed metals are known to be components of munitions (Alternatives for the Demilitarization of Conventional Munitions, 2019). Several time-series concentration patterns were observed for the 13 metals, which would be anticipated because 1) the waste stream was never exactly the same with different munitions containing different metals, and 2) wind speed and direction naturally vary over time, there was not a typical pattern of metals deposition with distance from the facility. Typically, concentrations were similar at the 1.2 mi and 9.0 mi sites, and several of the measured concentrations were above the 50th percentile of the county-level average PM_2.5_ metal concentrations where speciation monitoring data were available in 2022 for Al, Cr, Cu, Fe, Mg, Mn, Ni, Pb, and V based on data reported in the U.S. EPA’s Air Quality System (US EPA, 2022b). Al, Ba, Mg, and V exhibited high concentration spikes in summer 2022 from June to August, while the highest concentrations for Cr, Ni, and Sb were detected in November, 2022. Two relatively high concentration clusters of Cu, Fe, and Mn were observed in June and November, 2022. Cd concentrations increased throughout the sampling campaign, being almost non-detectable from April to June and reaching its highest levels in February, 2023. Pb concentrations were relatively consistent throughout the year with two notable spikes measured only at the 1.2 mi site in June and September. Pb concentrations were also consistently higher during most weeks at the 1.2 mi site compared with the 9.0 mi site. Zn concentrations remained relatively consistent throughout the entire sampling campaign. Distinct signals of Cr, Cu, Fe, Mn, Ni, and Sb were notable in November at the 1.2 mi site but not the 9.0 mi site.

Unlike PM_2.5_, concentrations of EPFRs did not decline substantially in the fall and winter, so that EPFRs comprised a greater share of the PM_2.5_ later in the 2022–2023 sampling period. For the first half of the sampling year, EPFRs concentrations at the two sites did not always follow the same trend, but the two sites’ time series were similar for the second half of the year. PM_2.5_ and EPFRs have low-moderate correlation in the first half of the sampling year, but correlations improve greatly for the second half of the year (Table 1). This reflects similar weekly variability in PM_2.5_ and EPFRs at both locations except for a few weeks. For example, peak PM_2.5_ concentrations occurred in June, while peak EPFR concentrations were observed in September (Fig. 2). Several factors may account for observed differences. The types of materials detonated during different periods may have produced elevated EPFR levels even with lower PM_2.5_ concentrations. During the first part of the year, moderate Pearson correlation of EPFRs with Pb was observed at the 1.2 mi site, and both Pb and Zn were moderately-highly correlated with EPFRs at the 9.0 mi site during this time. During the second half of the year, increased correlations of EPFRs with Pb, Al, Ba, Mn, Cu, and Mg were observed at both sites. Correlation between EPFRs and Zn dropped slightly at the 9.0 mi site compared with the 1.2 mi site in the second half of the year. Commonalities in correlations at the two sites suggest a common source at both sites even with potential explanations for some differences. Blockage of the plume by nearby trees may curtail some of the plume at the 1.2 mi site. A second source could have contributed to measurements at the 9.0 mi site, especially in the first part of the year when the EPFRs time series exhibited greater differences compared with later in the year. Presence of ultrafine particles in the sample could be correlated to concentrations of EPFRs and PM_2.5_ mass concentration (Table 1) yet contribute minimally to the PM_2.5_ mass concentration itself. A study on EPFR particle size distribution has reported that the highest levels of EPFRs were present in the PM fraction with aerodynamic diameter *<*1 μm (Yang et al., 2017). Additionally, EPFR concentrations are sensitive to variations in humidity (Nwosu et al., 2016; Pan et al., 2019).

Elastic Net models (Table 2) show that a one standard deviation-change in the significant metal (or EPFR for the PM_2.5_ model) may be associated with a one standard deviation-change in PM_2.5_ or EPFR concentration. For PM_2.5_, the full year of data produced significant coefficients for Zn, PM_2.5,−1_, Mg, V, Pb, Mn, Cu, EPFRs, and Al at the 1.2 mi site and largely agreed with the Pearson correlations (Table 1). The PM_2.5_ prediction model only produced a significant coefficient for EPFRs at the 9.0 mi site, with a coefficient that is more than 25 times the EPFR coefficient at the 1.2 mi site. However, no metals were significant for this model, which had the greatest shrinkage and minimized mean squared errors (MSE). Hence, PM_2.5_ time series for both sites were influenced by EPFRs likely generated by combustion, but only the site closest to the facility saw PM_2.5_ time series directly influenced by the metals (most notably Zn). Because the concentrations of all parameters were normalized by their standard deviations, the results do not reflect which compounds contribute the most mass. Rather, these indicate which compounds may drive changes over time. While Mg is abundant in soil in Central Louisiana (Smith et al., 2013) and can be a potential source of PM_2.5_, heavy metals including Zn, Cd, Pb, and Mn are not typically found in notable quantities in rural areas (Smith et al., 2013).

For EPFRs, the full year of time series data produced significant coefficients for EPFR_-1_, Zn, Cd, Pb, Ni, Cr, and Mn as significant predictors of EPFRs concentrations at the 1.2 mi site. For the 9.0 mi site, EPFR_-1_, Zn, Sb, Pb, and Ni were significant predictors of EPFRs concentrations. These observations support previous research, which has shown that EPFR formation is dependent on interaction between pollutant and transition metal-surface (Dellinger et al., 2007; Khachatryan et al., 2011; Lomnicki et al., 2008b). With consistent findings in the Pearson correlation analysis and the elastic net model, there is the greatest confidence in the positive Zn results compared with all other heavy metals. This strong relationship suggests that Zn plays a significant role in EPFR formation in these samples, supported by previous research showing that Zn is a suitable surface for the electron-transfer to take place (Sakr et al., 2019). Vejerano et al. (2018) offers band-bending (Vejerano et al., 2018), or a transfer of electrons when two surfaces are in contact (Zhang and Yates, 2012), as a potential mechanistic explanation for EPFR formation with Zn. Additionally, these results are consistent with previous works, as similar strong correlations between Zn and EPFR concentrations have been observed on field-collected samples (Feng et al., 2022; Lard et al., 2025). Zn is commonly used as a metal plating material in munitions (Lima et al., 2011). Although Zn is an essential dietary nutrient, inhalation of Zn in particulate matter can cause respiratory distress (Cooper, 2008).

To our knowledge, this is the first year-long study of air pollutant composition in the vicinity of an open-burn munitions source. The data presented provides a comprehensive picture of the PM_2.5_ components to which Colfax residents are exposed. However, it is important to acknowledge limitations of the study. Only two sampling sites were established for comparison, and a true upwind site could not be identified given the high proportion of calm winds and the high correlation between the PM_2.5_ time series at both sites (ρ = 0.84). Without complete burn log information being made available, it was not possible to conduct a comprehensive source apportionment or accurately identify other potential sources of the investigated pollutants. Replicated samples were not available for each collection period at both sites. Limited numbers of PCDD/Fs analyses could not provide whole year trend and a better correlation with other analytes. Reliance on elastic net regression methods to identify relationships between the metals and EPFRs creates the potential for overinterpretation of our results, because these methods are inherently designed for screening (Tibshirani, 1996, 2011; Zou and Hastie, 2005). The elastic net models do not describe definitive relationships of PM_2.5_ and EPFRs with the metals. Rather, they illustrate which metals may have been most influential in driving changes in the PM_2.5_ and EPFR data set across the sampling period. Lack of a significant coefficient for a given metal does not mean that the compound was not present.

## 4. Conclusions

This study, as the first year-long effort to collect and analyze ambient air pollutants in The Rock and Colfax community, provided community members with quantitative results about the pollutants they were exposed to, helping to substantiate their experiences and understand their exposure risks and how exposures may relate to health issues experienced by many members of the community. Our measurements consistently detected PM_2.5_, EPFRs, dioxins and furans, with several heavy metals (Mn, Al, Mg, Fe, V) were routinely observed well above their national averages. Correlation and elastic net regression analyses pointed to Zn having a strong relationship with both PM_2.5_ and EPFRs. As a component of munitions housing, airborne Zn in PM_2.5_ has been demonstrated to play a role in EPFR formation (Sakr et al., 2019; Vejerano et al., 2018) and unsurprisingly is significantly associated with EPFR concentration. Future work will focus on examining the spatial variability of pollutants by incorporating lower-cost sampling methods to cover a larger area. This information will help us integrate spatial and temporal results, thus allowing for a more comprehensive evaluation and prediction of local exposure. We hope these efforts will enhance community engagement, inform decision-making and provide some spatial data that can be used in other studies evaluating the OB/OD exposure risks.

As a community-engaged study, this work was designed and developed to address residents’ concerns, with data reported quarterly through a custom community reporting tool (Sauermilch et al., 2025). During most visits to Colfax, the research team has held community meetings to present preliminary study design for community input and approval, to present results, to answer community members’ questions, and to respond to community members’ evolving needs regarding data collection and interpretation. Reports containing presented data have been created and distributed among community members during these meetings, during sampling trips at residents’ homes, through door-to-door dissemination of results, and between community members themselves. The implementation of this environmental health communication approach has supported community members in advancing environmental justice efforts, because this information has been used by the community members for advocacy with regard to efforts to stop OB/OD operations. The research team has also used these results to educate policymakers on the environmental conditions in Colfax and the health implications of these conditions for the residents.

## Supplementary Material

Guo et al 2025 SI

## Figures and Tables

**Table 1:** Pearson correlation of EPFRs and PM_2.5_ sampled with HiVols at 1.2 mi and 9.0 mi from the TT facility with particle surface area for particles smaller than 0.3 μm, 0.5 μm, 1.0 μm, and 2.5 μm and metals in PM_2.5_ using a 1-week averaging time. Surface area data were bias corrected for temperature and relative humidity before calculating concentrations. “NA” denotes that data were not available for a given parameter, site, and time frame.

	1.2 mi						9.0 mi					
	Year-long		Spring-Summer		Fall-Winter		Year-long		Spring-Summer		Fall-Winter	
	PM_2.5_	EPFRs	PM_2.5_	EPFRs	PM_2.5_	EPFRs	PM_2.5_	EPFRs	PM_2.5_	EPFRs	PM_2.5_	EPFRs

PM_2.5_	1.00	−0.016	1.00	0.19	1.00	0.64	1.00	0.40	1.00	0.47	1.00	0.76
EPFRs	−0.016	1.00	0.19	1.00	0.64	1.00	0.40	1.00	0.47	1.00	0.76	1.00
d_p_ < 0.3 μm	0.76	0.010	0.51	0.028	0.85	0.48	0.72	0.53	0.78	0.45	0.79	0.61
d_p_ < 0.5 μm	0.76	0.00021	0.53	0.040	0.85	0.48	0.72	0.53	0.78	0.45	0.79	0.61
d_p_ < 1.0 μm	0.71	0.087	0.80	0.23	0.77	0.32	0.60	0.36	0.76	0.35	0.63	0.37
d_p_ < 2.5 μm	0.64	−0.071	0.78	0.11	0.50	0.015	0.21	0.19	0.47	0.062	0.29	0.040
Al	0.61	−0.19	0.64	−0.18	0.73	0.47	0.52	−0.095	0.47	0.090	0.73	0.53
Ba	0.62	−0.091	0.69	−0.16	0.48	0.59	0.59	0.022	0.63	0.12	0.47	0.47
Cd	−0.25	0.27	0.11	0.12	−0.051	−0.097	−0.20	−0.23	0.39	0.28	−0.33	−0.43
Cr	−0.015	0.046	0.41	−0.25	0.21	−0.093	−0.015	0.12	0.72	−0.011	0.044	0.029
Cu	0.24	0.18	0.38	0.074	0.37	0.21	0.39	0.37	0.48	0.32	0.40	0.44
Fe	0.42	−0.094	0.65	−0.18	0.33	−0.019	0.44	0.019	0.53	0.11	0.29	0.19
Mg	0.72	−0.20	0.70	−0.12	0.65	0.29	0.58	−0.0094	0.45	0.090	0.69	0.38
Mn	0.62	−0.0028	0.76	−0.029	0.53	0.20	0.68	0.37	0.68	0.21	0.68	0.50
Ni	−0.0036	0.057	0.65	−0.10	0.23	−0.083	0.14	0.35	0.48	−0.097	0.30	0.29
Pb	0.12	0.25	−0.23	0.37	0.44	0.70	0.38	0.61	0.52	0.35	0.53	0.71
Sb	−0.033	0.036	NA	NA	0.19	−0.12	−0.046	0.36	−0.066	0.39	0.053	0.28
V	0.69	−0.23	0.67	0.0087	0.52	−0.056	0.44	−0.027	0.29	0.015	0.42	0.013
Zn	0.55	0.38	0.58	0.17	0.81	0.71	0.65	0.57	0.76	0.65	0.73	0.55

**Table 2:** Elastic net model results for full year of sampling for PM_2.5_ and EPFRs at the 1.2 mi and 9.0 mi sampling sites. “NA” denotes that the compound (EPFR) was not included in that model. Lag-1 term indicates PM_25_ from the previous week (PM_2.5,−1_) for the PM_2.5_ model and EPFRs from the previous week (EPFR_−1_) for the EPFRs model. A dash indicates that the shrinkage algorithm did not select the variable. Model selection statistics are provided in the bottom three rows of the table.

	1.2 mi		9.0 mi	
	PM_2.5_	EPFRs	PM_2.5_	EPFRs

Intercept	−0.0303	−0.113	0.0677	0.342
Lag-1 term	0.246	0.522	−	0.315
EPFR	0.0370	NA	0.971	NA
Al	0.0315	−	−	−
Ba	−	−	−	−
Cd	−	0.148	−	−
Cr	−	0.00258	−	−
Cu	0.0497	−	−	−
Fe	−	−	−	−
Mg	0.215	−	−	−
Mn	0.101	0.00121	−	−
Ni	−	0.0245	−	0.0122
Pb	0.104	0.0468	−	0.0297
Sb	−	−	−	0.0707
V	0.198	−	−	−
Zn	0.293	0.238	−	0.275
*α*	*0*	*0*	*1*	*0.5*
*λ*	*0.0800*	*0.165*	*0.0291*	*0.263*
*MSE*	*0.142*	*0.504*	*0.000976*	*0.686*

## Appendix A. Supplementary data

Supplementary data to this article can be found online at https://doi.org/10.1016/j.apr.2025.102769.
