# Supplementary material for: Persistent chemicals in particulate matter (PM) near a hazardous waste thermal treatment facility
Source: Atmos Pollut Res. Author manuscript; Available in PMC 2025 Nov 26. (PMC12646043; doi:10.1016/j.apr.2025.102769)
Supplement: Guo et al 2025 SI [file NIHMS2123273-supplement-Guo_et_al_2025_SI.docx]

**Supporting Information**

**Persistent chemicals in particulate matter (PM) near a hazardous waste thermal treatment facility**

*Chuqi Guo^1^, Martine E. Mathieu-Campbell^2,3^, Thomas Blanchard^4^, Lavrent Khachatryan^5^, Md Abdullah Al-Mamun^6^, Qingzhao Yu^6^, Myron Lard^1^, Oluwafeyikemi Ogunmusi^7^, Brenda Vallee^8^, Wilma Subra^9^, Iriel Edwards^10^, David Malone^11^, Slawo Lomnicki^7^, Stephania A. Cormier^12^, Jennifer Richmond-Bryant^1,2,*^*

^1^Department of Forestry and Environmental Resources, North Carolina State University, Raleigh, NC 27695, USA

^2^Center for Geospatial Analytics, North Carolina State University, Raleigh, NC 27695, USA

^3^Department of Biostatistics and Bioinformatics, Rollins School of Public Health, Emory University, Atlanta, GA 30322, USA

^4^Department of Oceanography and Coastal Sciences, Louisiana State University and A&M College, Baton Rouge, LA 70803, USA

^5^Department of Chemistry, Louisiana State University and A&M College, Baton Rouge, LA 70803, USA

^6^School of Public Health, Louisiana State University Health Sciences Center, New Orleans, LA 70112, USA

^7^Department of Environmental Sciences, Louisiana State University and A&M College, Baton Rouge, LA 70803, USA

^8^Central Louisiana Coalition for a Clean and Healthy Environment, Colfax, LA 71417, USA

^9^Louisiana Environmental Action Network, Baton Rouge, LA 70806, USA

^10^Shreveport Green, Shreveport, LA 71104, USA

^11^Louisiana Department of Wildlife and Fisheries Region 3, Pineville, LA 71360, USA

^12^Department of Biological Sciences, Louisiana State University and A&M College and Pennington Biomedical Research Institute, Baton Rouge, LA 70803, USA

Number of pages: 10

Number of tables: 3

Number of figures: 5

Corresponding author email: [jrbryan3@ncsu.edu](mailto:jrbryan3@ncsu.edu)

Text S1. Metals analysis and quality assurance/quality control

All metal analyses in this study were performed using an Agilent 5800 ICP-OES (Agilent Technologies, USA) equipped with an easy-fit 1.8 mm torch one piece and a 1.8 mm ID injector (Agilent Technologies, USA), a double-pass glass cyclonic spray chamber (Agilent Technologies, USA), and a glass Seaspray nebulizer U-series (Agilent Technologies, USA). Samples were introduced using an Agilent SPS-4 autosampler (Agilent Technologies, USA) with white/white PVC tubing for sample introduction, blue/blue for the waste drain line and yellow/orange for the 10 ppm Yttrium (Y) internal standard. Indium (In) and Neodymium (Nd) were added to the digestion tubes to a final concentration of 1 ppm as method recovery standards at the beginning of the digestion process. Certified reference standards (Waters ERA, Golden CO) were run every 10 samples to ensure optimal instrument performance. A 10 ppm Yttrium internal standard was used to correct for the instrumental drifts and non-spectral interferences. Matrix spikes were analyzed every 10 samples and were within 90-110 % recovery. Method detection limits (MDL) for each metal were determined using combined methods of three times standard deviation from seven (7) replicates of spiked filter blanks treated the same way as the samples, and the concentration levels that corresponds to an instrument signal-to-noise ratio in the range of 3 to 5 (US EPA, 2016).

Text S2. Time-series analysis methods

Time-series analysis of the weekly data entailed first graphing autocorrelation and partial autocorrelation functions available through the R statistical software using the *stats* library to confirm the time series structure. The data for each parameter (PM_2.5_, EPFRs, and all metals) were first normalized by standard deviation so that time-series analysis and regression coefficients would be on the same scale. Autoregression Integrated Moving Average (ARIMA) models were fit to the weekly data for PM_2.5_, EPFRs, and each metal component based on the dominant timescales, because autoregression can inflate the mean squared error of the model^80^. The majority of ARIMA models displayed a lag-1 autoregressive function.

Table S1. Quantity of ammonium perchlorates declared in the burn logs coinciding with the sample collection periods

| Collection | Declared ammonium perchlorate (pounds) |
| --- | --- |
| 04/08/22-04/15/22 | Not declared (Class 1.X destroyed) |
| 06/17/22-06/24/22 | 520 |
| 07/01/22-07/15/22 | 1170 |
| 09/17/22-09/24/22 | Not declared/no burn logs available |
| 11/19/22-11/26/22 | 300 |
| 12/16/22-12/26/22 | 331 |

Table S2. Method detection limits (MDL) for QF samples (mg/L)

| **Metal** | **Ag** | **Al** | **As** | **Ba** | **Be** | **Cd** | **Co** | **Cr** | **Cu** |
| --- | --- | --- | --- | --- | --- | --- | --- | --- | --- |
| **MDL** | 0.0052 | 0.0172 | 0.1000 | 0.0070 | 0.0013 | 0.0014 | 0.0500 | 0.0106 | 0.0200 |
|  |  |  |  |  |  |  |  |  |  |
| **Metal** | **Fe** | **Mg** | **Mn** | **Ni** | **Pb** | **Sb** | **V** | **W** | **Zn** |
| **MDL** | 0.0378 | 0.0010 | 0.0100 | 0.0043 | 0.0300 | 0.0500 | 0.0100 | 0.0500 | 0.0033 |

Table S3. The 50^th^ percentile of the average PM_2.5_ metal concentrations where speciation monitoring data were available in 2022(U.S. Environmental Protection Agency, n.d.)

| **50^th^ percentile across estimates for (ng/m3):** | **Al** | **Ba** | **Cd** | **Cr** | **Cu** | **Fe** | **Mg** | **Mn** | **Ni** | **Pb** | **Sb** | **V** | **Zn** |
| --- | --- | --- | --- | --- | --- | --- | --- | --- | --- | --- | --- | --- | --- |
| **US avg** | 39.5 | 6.39 | 1.11 | 0.534 | 1.08 | 52 | 18.4 | 1.25 | 0.221 | 1.29 | 1.59 | 0.068 | 5.72 |
| **US rural avg** | 38.5 | 3.51 | 0.863 | 0.009 | 0.166 | 32.8 | 15.5 | 0.923 | 0 | 0.287 | 0.00031 | 0.009 | 1.47 |
| **Southeast avg** | 42.9 | 4.41 | 0.965 | 1.03 | 1.42 | 53.5 | 16.9 | 1.03 | 0.465 | 1.95 | 1.85 | 0.093 | 7.34 |
| **LA avg** | 80.4 | 3.05 | 0.703 | 0.905 | 1.05 | 72.7 | 30 | 1.82 | 0.553 | 1.25 | 1.9 | 0.399 | 6.16 |

Figure S1. Aerial photograph of Colfax, LA and points north, including The Rock community and the TT facility (Richmond-Bryant et al., 2024).

-
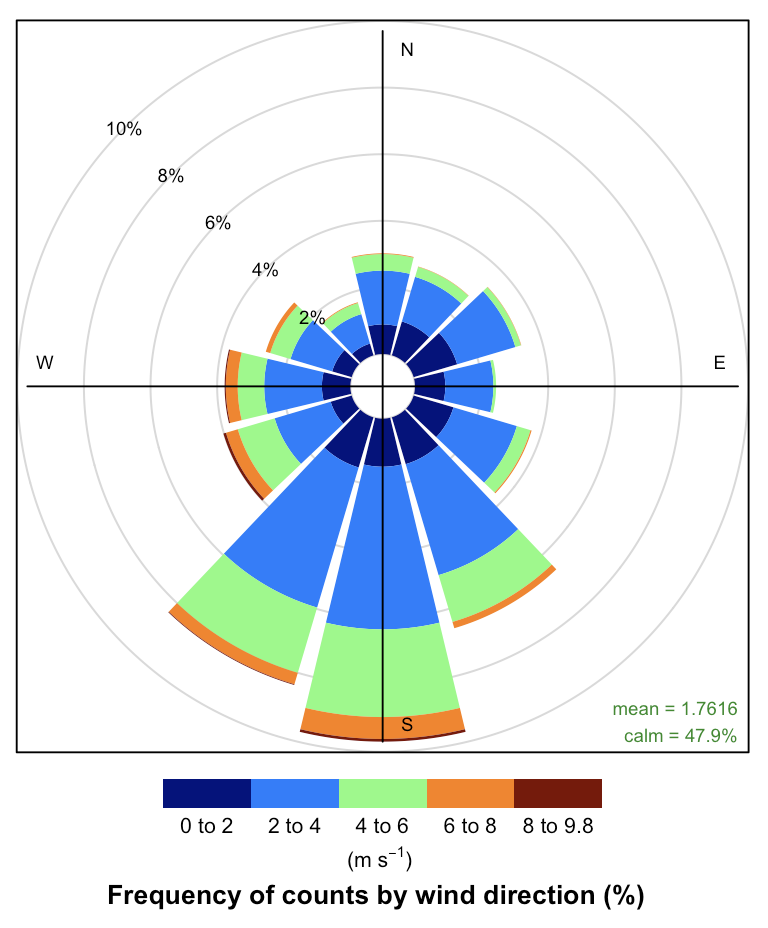


Figure S2. Wind rose for the 1-year sampling campaign.

Figure S3. Seasonal wind roses.

Figure S4. Monthly wind roses.

Figure S5. The contribution of congeners, calculated as the product of the TEF and the congener’s concentration, to the TEQ in each collection.

**References**

Richmond-Bryant, J., Odera, M., Subra, W., Vallee, B., Rivers III, L., Kelley, B., Cramer, J.A., Wilson, A., Tran, J., Beckham, T., Irving, J., Reams, M., 2024. Oral histories document community mobilisation to participate in decision-making regarding a hazardous waste thermal treatment facility. Local Environment 29, 57–73. https://doi.org/10.1080/13549839.2023.2249498

U.S. Environmental Protection Agency, n.d. Table of Annual Summary Data, 2022 [WWW Document]. URL https://aqs.epa.gov/aqsweb/airdata/annual_conc_by_monitor_2022.zip (accessed 8.15.24).

US EPA, 2016. Definition and Procedure for the Determination of the Method Detection Limit, Revision 2.
